# An Updated View of the *Trypanosoma cruzi* Life Cycle: Intervention Points for an Effective Treatment

**DOI:** 10.1021/acsinfecdis.2c00123

**Published:** 2022-06-02

**Authors:** Javier Martín-Escolano, Clotilde Marín, María J. Rosales, Anastasios D. Tsaousis, Encarnación Medina-Carmona, Rubén Martín-Escolano

**Affiliations:** † Unit of Infectious Diseases, Microbiology and Preventive Medicine, Institute of Biomedicine of Seville (IBiS), University Hospital Virgen del Rocío/CSIC/University of Seville, E41013 Seville, Spain; ‡ Department of Parasitology, University of Granada, Severo Ochoa s/n, 18071 Granada, Spain; § Laboratory of Molecular & Evolutionary Parasitology, RAPID group, School of Biosciences, University of Kent, Canterbury CT2 7NJ, U.K.; ∥ Department of Physical Chemistry, University of Granada, 18071 Granada, Spain; ⊥ School of Biosciences, University of Kent, Canterbury CT2 7NJ, U.K.

**Keywords:** Chagas disease, drug discovery, evolution model, genetic diversity, life cycle, morphological forms, target product profile, tropism, *Trypanosoma cruzi*

## Abstract

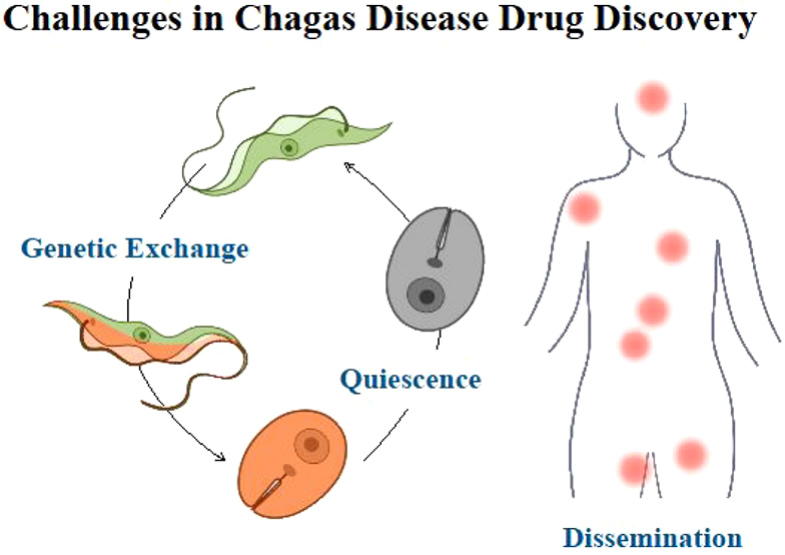

Chagas disease (CD) is a parasitic, systemic, chronic, and often fatal illness caused by infection with the protozoan *Trypanosoma cruzi*. The World Health Organization classifies CD as the most prevalent of poverty-promoting neglected tropical diseases, the most important parasitic one, and the third most infectious disease in Latin America. Currently, CD is a global public health issue that affects 6–8 million people. However, the current approved treatments are limited to two nitroheterocyclic drugs developed more than 50 years ago. Many efforts have been made in recent decades to find new therapies, but our limited understanding of the infection process, pathology development, and long-term nature of this disease has made it impossible to develop new drugs, effective treatment, or vaccines. This Review aims to provide a comprehensive update on our understanding of the current life cycle, new morphological forms, and genetic diversity of *T. cruzi*, as well as identify intervention points in the life cycle where new drugs and treatments could achieve a parasitic cure.

Chagas disease (CD), also known as Chagas-Mazza or American trypanosomiasis, is a parasitic, systemic, chronic, and life-threatening disease caused mainly by infection with the triatomine-transmitted protozoan parasite *Trypanosoma cruzi*.^1^ CD is the most important parasitic disease in Latin America since it is one of the most frequently occurring causes of heart failure, it causes the loss of around 752 thousand working days due to premature deaths, and it causes US$1–2 billion in productivity losses.^2,3^ The World Health Organization (WHO) has classified CD as being among the 20 Neglected Tropical Diseases (NTDs), estimating that 6–8 million people are infected worldwide: 28 000 new infections and 14 000–50 000 deaths occur every year, and 70–100 million people are at risk of infection.^4−7^ In 2020, drawing on the expertise of clinicians, researchers, implementation science experts, and patients, the WHO introduced a roadmap with the following objectives: verifying the interruption of vector-borne transmission, verifying the interruption of transmission by transfusions and organ transplants, eliminating congenital CD, and broadening antiparasite treatment coverage by 75% regarding the population at risk.^1^

CD was discovered by Carlos Ribeiro Justiniano Chagas in 1909.^4,8^ This Brazilian medical researcher also identified the etiological agent *Trypanosoma cruzi*, the hosts, the triatomine vectors (mainly *Triatoma infestans*, *Rhodnius prolixus*, and *Triatoma dimidiata*), and the different developmental stages of the parasite, as well as the clinical aspects of CD and its epidemiology. This period is considered the founding stage.^8−10^ In the 1930s, Salvador Mazza confirmed the endemic nature of CD and defined the anatomical–clinical stages of the disease.^10^ However, it was not until 1960 that nifurtimox (NFX), the first drug to combat CD, was developed. Subsequently, benznidazole (BZN) was the second and last drug developed, in 1972. Despite the fact that the WHO classifies CD as the most prevalent of the poverty-caused and poverty-promoting NTDs, the most important parasitic disease, and the third most spread infectious disease in Latin America,^5,11,12^ currently approved treatments are limited to these two nitroheterocyclic drugs developed more than 50 years ago. Serious drawbacks include long treatment periods, toxic side effects, and in many cases failures in treatment.^13−15^

CD presents two clinical phases, the pathology of which is modulated by (1) complex genetic interactions among the host and the parasite, (2) environmental and social factors, and (3) mixed infections, reactivations, and re-infections.^16^ The acute phase is characterized by high parasitemia, often accompanied by systemic symptoms, such as fever, headache, and diarrhea, among others, and occasionally lymphadenopathy, hepatomegaly, splenomegaly, myocarditis, and meningoencephalitis.^12,16−18^ Subsequently, most infected people continue with the asymptomatic phase, but around 30% of infected people progress to the chronic phase, in which several organs affected as a result of a severe inflammatory immune response leading to irreversible cell damage, with Chagas cardiomyopathy being the main cause of fatality.^12,17−19^

Many efforts have been made in recent decades to develop new treatments, but the biology of the infection, the complex pathology, and the long-term nature of CD have made it impossible to find new drugs and/or effective treatments. Amlodipine, atenolol, bisoprolol, clioquinol, defibrotide, ivermectin, meglumine, metformin, miltefosine, paromomycin, pentamidine, pyrimethamine, and tinidazole are a few examples of compounds with trypanocidal activity, but the results have been inconclusive in the clinic.^20,21^ Alternatively, an immune therapy that will control *T. cruzi* transmission and chronic CD is urgently required. It is widely accepted that a vaccine should include antigens targeting all morphological stages of *T. cruzi* and should be useful as a prophylactic and therapeutic vaccine. Significant efforts associated with developing vaccines have also been made, leading to some promising results in animal models. Immunogens, adjuvants, and DNA-based vaccines have been used in the search for vaccines candidates, but a vaccine is not yet available.^22,23^ Recently, a new RTS,S/AS01 vaccine has been made available for preventing malaria infection,^24−27^ which could spearhead future developments against CD as well.

In recent years, progress has been made regarding *T. cruzi* stage forms and its life cycle: recent studies have (a) discovered new morphological forms with a major impact on the new view of the life cycle of *T. cruzi*—highlighting those of high clinical impact—and (b) identified key stages that allow genetic exchange between *T. cruzi* strains, exponentially increasing the genetic variability of this parasite. On the one hand, these findings help the understanding of this disease and lead the research for new treatments. On the other hand, they are the challenges that make it more difficult to develop an effective treatment to achieve a parasitic cure. This Review aims at providing an update regarding the current view of the life cycle, new morphological forms involved, and the genetic diversity/genetic exchange of *T. cruzi*.

## Classical Life Cycle

The classical version of the life cycle of *T. cruzi* involves two hosts and four stages. The infection of a mammalian host begins with the non-dividing metacyclic trypomastigotes present in the excreta of the blood-feeding triatomine vector, which penetrate through the vector bite wound or a variety of mucosal membranes. Initially, they bind to receptors on a wide range of phagocytic and non-phagocytic nucleated cells and enter a membrane-bound vacuole called a parasitophorous vacuole (PV). Upon entry, the parasites differentiate into small round-shaped amastigotes and escape the PV into the cell cytoplasm, where the morphologic transformation is completed, including flagellar involution. The amastigotes re-enter the cell cycle and proliferate by binary fission until the cells fill with these replicative forms. At this point, the amastigotes elongate, reacquire the long flagella, and differentiate into non-replicative trypomastigotes. The trypomastigotes are forms that show continuous and intense movement, while they induce lysis of the host cell membrane. Once the trypomastigotes are released, they can invade adjacent cells or enter the blood and lymph and disseminate. These bloodstream trypomastigotes (BTs) can be taken up by triatomine vectors, and in the vector midgut, parasites become epimastigotes and proliferate. Finally, the epimastigotes migrate to the vector hindgut and attach to the waxy gut cuticle by their flagella to differentiate into metacyclic trypomastigotes.^28,29^

## New Morphological Forms

Further research^29−38^ has revealed that the classical view is rather superficial and that the process in mammalian hosts is certainly more complex. We will take a historical approach to disclose these revisions.

In 1963, a pleomorphic population made up of a mixture of two basic morphologies, slender and broad BTs, was identified in the blood: slender forms are more able to enter tissue cells, are more infectious (being capable of infecting both by penetration and by phagocytosis), determine earlier parasitemia, and are more sensitive to circulating antibodies; in turn, broad forms remain longer in the bloodstream, are less infectious (being capable of infecting only through phagocytosis), develop later parasitemia, and are more resistant to antibodies.^30,31^ Moreover, slender and broad forms exhibit different localization (tropism):^32,33^ slender forms mainly infect mononuclear phagocytic system cells, showing tropism for spleen, liver, and bone marrow, whereas broad forms exhibit tropism for cardiac, skeletal, and smooth muscle cells. Depending on the relative proportion of both forms of a certain strain, its biological behavior can vary, thus affecting the infection outcome in the host. The relative proportion of slender and broad forms is *T. cruzi* strain-dependent; thus, the biological behavior, the outcome of the infection, and the efficacy of treatments vary. In addition, extracellular differentiation of BTs to amastigotes was also observed (trypomastigotes are programmed to develop into amastigotes whether or not they enter cells^34^), and a mixture of these three forms may be present in the blood of infected mammalian hosts.^29^

In 2003, intracellular epimastigote-like forms were reported, although it is unclear whether this form represents an obligate intracellular stage of the life cycle or is simply an intermediate in the transition from amastigotes to trypomastigotes.^29,35^

In 2014, a new intracellular morphology called zoid was identified. This form results from the initial differentiation from the metacyclic trypomastigotes through asymmetric cell division, resulting in one amastigote and one zoid. This zoid is a cell with kinetoplast but no nucleus, which quickly dies and is degraded by the host cell.^36^

A new, clinically noteworthy finding was reported in 2017. It was noted that some amastigotes may become metabolically quiescent (so-called quiescent or dormant amastigotes), an important fact concerning drug resistance in Chagas disease (CD). These forms can reside long term in chronically infected tissues in mammalian hosts^35,37^ and are able to spontaneously resume cell cycle and re-establish infection, even after treatment. The existence of an adaptive difference between *T. cruzi* strains to induce dormancy has been suggested.^38^ Dormancy is a state involved in resistance to non-optimal environmental conditions, and it has been reported in several organisms, ranging from fungi^39^ and bacteria^40^ to other protozoa parasites—such as certain *Plasmodium* spp.^41^ and *Toxoplasma* spp.^42^—and cancer cells.^43^ Dormancy is recognized as a particular stage associated with disease recurrence and drug resistance. In any case, the mechanisms of dormancy in *T. cruzi* are not fully identified yet, but homologous recombination is essential in this phenomenon.^38,44^ In summary, studies on the mechanism of dormancy should be addressed in order to therapeutically override it.

The process in the triatomine vector is also more complex. Bloodstream amastigotes from mammalian hosts differentiate into forms with short flagella in the gut of triatomines. These forms are called sphaeromastigotes, although they probably represent intermediates in the transition to epimastigotes.^29^ Alternatively, metacyclic trypomastigotes have been shown to have the capacity to differentiate into epimastigote-like forms. These forms exhibit a distinct proteomic fingerprint and are capable of invading mammalian host cells to initiate a new infection.^45^

Table 1 summarizes the main morphological forms of *T. cruzi* in both mammalian hosts and triatomine vectors.

**Table 1:** Main *Trypanosoma cruzi* Morphological Forms in Both Mammalian Hosts and Triatomine Vectors

morphological form	host	stage (mammalian host)^a^	location	extracellular/intracellular	replicative/non-replicative	infectious/non-infectious	response to current drugs	differentiate into	refs
metacyclic trypomastigote	triatomine/mammalian	early acute stage (infective form)	hindgut and excreta/blood	extracellular	non-replicative	infectious	—	amastigote	(28, 29, 45)
amastigote	mammalian/triatomine	acute and chronic stages	target organs and blood/stomach	intracellular/extracellular	replicative/non-replicative	infectious	sensitive (mostly)	bloodstream trypomastigote/sphaeromastigote	(28, 29)
zoid	mammalian	quickly degraded	—	intracellular	non-replicative	non-infectious	—	*degraded*	(36)
quiescent/dormant amastigote	mammalian	indeterminate stage	target organs	intracellular	non-replicative	non-infectious	resistant	amastigote	(35, 37, 38)
bloodstream trypomastigote (BT)									
slender BT	mammalian/triatomine	acute and chronic stages	blood and lymph/stomach	extracellular	non-replicative	(more) infectious	sensitive (mostly)	amastigote/epimastigote	(28−34)
broad BT	mammalian/triatomine	acute and chronic stages	blood and lymph/stomach	extracellular	non-replicative	(less) infectious	sensitive (mostly)	amastigote/epimastigote	(28−34)
epimastigote	triatomine/mammalian (unclear)	—	midgut/target organs (unclear)	extracellular/intracellular (unclear)	replicative	non-infectious^b^	—	metacyclic trypomastigote/trypomastigote	(28, 29, 35, 45)
sphaeromastigotes	triatomine	—	midgut	extracellular	non- replicative	non-infectious	—	epimastigote	(29)

a It should be noted that the spatiotemporal dynamic of the parasite during the chronic stage is changeable.

b They can be infective according to some authors.

## Genetic Diversity and New Evolution Model

Knowledge on the genetic diversity of *T. cruzi* is relatively recent.^46−48^ Although it is not an obvious aspect of the life cycle of the parasite, such as the identification of new morphologies, it is directly related to the biology of infection and the tropism of *T. cruzi* in mammalian hosts. Hence, it is a crucial aspect to consider in the development of an effective treatment.

The first report on the important variation in drug susceptibility among *T. cruzi* strains was published in 1976.^46^ In 1982, high genetic intraspecific diversity of *T. cruzi* was reported, showing a difference up to 40% in both nuclear and kinetoplast DNA content between strains.^49^ Such a difference would be equivalent to 73 Mb of DNA, an amazing finding for a population of the same species.^47^ In 1999, phylogenetic reconstructions by comparative analysis based on ribosomal DNA (rDNA) sequences suggested that *T. cruzi* strains diverged about 100 million years ago,^50^ and two major lineages were described: *T. cruzi* I, which is associated with human disease in all endemic countries north of the Amazon basin, and *T. cruzi* II, which predominates in the southern cone countries of South America and is subdivided into five discrete typing units (DTUs): IIa, IIb, IIc, IId, and IIe.^48,51,52^ In 2006, the existence of a third lineage (*T. cruzi* III) was reported.^53^

Finally, an expert committee considered previous studies based on the pattern of genetic, biochemical, and biological markers and proposed in 2009 a minimum of six genetic lineages or DTUs (TcI–TcVI),^54^ with a seventh proposed (TcBat) related to TcI.^55,56^ Analyses from genealogies of mitochondrial sequences identified in 2016 three clades that hold a correlation with the DTUs: clade A corresponds to TcI; clade B to TcIII, TcIV, TcV, and TcVI; and clade C to TcII.^57^

Since 2001, several articles have reported natural and habitual recombination in *T. cruzi*,^47,58−61^ as well as evidence that hybridization and genetic exchange are frequent between the dividing amastigotes.^62^ Moreover, similarly to the observation in other trypanosomatids, a genetic exchange among the epimastigotes localized in the gut and those attached to perimicrovillar membranes would be expected to occur in the triatomine vectors.^56,63,64^*T. cruzi* has been considered a clonal organism whose epimastigotes and amastigotes replicate by binary fission, and new clones evolve with the accumulation of discrete mutations. Currently, the traditional paradigm of the clonal evolution is challenged: recombination and genetic exchange have contributed to the present parasite population structures and to the evolution of distinct *T. cruzi* subgroups.^65^

In 2005, in an attempt to open up prospects for the development of novel diagnostic and therapeutic techniques, the whole genome of the *T. cruzi* CL Brener strain was sequenced.^66^ Currently, there are several reports of wide divergence in the susceptibility of the current treatments to different *T. cruzi* strains, independently of the mitochondrial nitroreductase sequence (enzyme required to activate current prodrugs, BZN and NFX). This fact implies that this susceptibility must be associated with additional genetic factors.^67−69^ These additional factors are most likely linked to the strain-dependent characteristics discussed above.

Currently, it is widely known that some DTUs are hybrids originating from genetic exchange events which occurred in the past, although it is still unknown whether one or more hybridization episodes happened in the history of this parasite.^70^ Recent data show that *T. cruzi* does reproduce sexually at high frequency via a mechanism consistent with classic meiosis, which may continue to transform contemporary disease cycles.^71^ This should give new impulse to the search for the site of genetic exchange within the host or vector to improve our ability to discern the genetic bases of virulence and drug resistance to treat and control CD.

In summary, it can be stated that *T. cruzi* belongs to a heterogeneous species consisting of a pool of strains and lineages that circulate among vectors and mammalian hosts. This heterogeneity could explain the geographical differences in disease pathology, morbidity, mortality, and treatment efficacy. However, no definitive correlation between disease severity and parasite lineage has been established,^12^ and this remains an area of great research interest. For example, certain lineages are more frequently associated with severe chronic CD.^72,73^

## Current Life Cycle

*T. cruzi* is a heteroxenic protozoan that fully embodies the characteristics of a successful parasite: it is maintained in nature by numerous vector species and mammalian host species distributed in most biomes and habitats in the Americas, such as marsupials, rodents, bats, armadillos, ranging carnivores, birds, domestic animals, and primates.^74^*T. cruzi* undergoes changes in morphology, nuclear shape, chromatin remodelling, gene expression, metabolism, mitochondrial DNA rearrangement, and relative volume alterations of the kinetoplast, reservosome, and lipid bodies, among others.^75,76^

As stated above, further research has revealed that the classical view is rather superficial and that the actual life cycle is certainly more complex. *T. cruzi* is a very heterogeneous species, it shows an extensive variety of morphological forms, and it is able to reach a wide variety of tissues in the chronic stage, much wider than classically known: adipose tissue, bladder, bone marrow, brain, heart, kidney, large intestine, liver, lung, lymph nodes, mesenteric tissue, muscle, esophagus, placenta, small intestine, skin, spleen, and stomach.^77−81^ In addition, parasites can become widely disseminated in chronic CD after reactivation in immunocompromised patients. Even the spatiotemporal dynamic of the parasite during the chronic stage is changeable, with foci that appear/disappear over the course of even a single day.^82^ Tropism is a crucial part of parasite development, but it is not just an intermittent feature of the life cycle. In fact, it is actually linked to key clinical phenotypes.^83^

**Figure 1:**
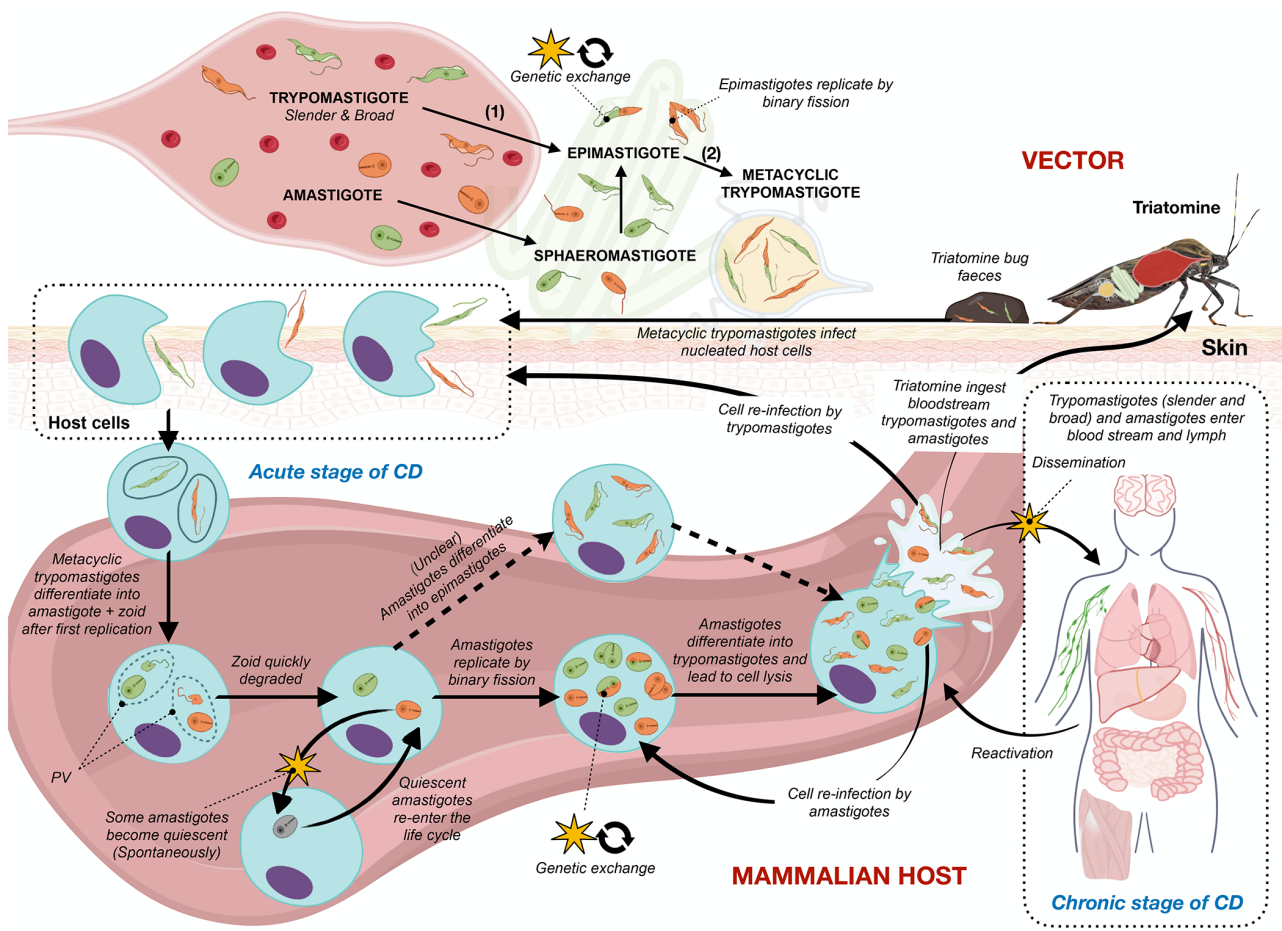
*Trypanosoma cruzi* life cycle and key challenges for the development of effective treatments for Chagas disease. (1) Trypomastigotes migrate to the midgut and differentiate into epimastigotes. (2) Epimastigotes migrate to the hindgut and differentiate into metacyclic trypomastigotes. Abbreviations: CD, Chagas disease; PV, parasitophorous vacuole. The green and orange colors of the parasites represent two different strains and the stages at which genetic exchange occurs.

Figure 1 summarizes the progress made in recent years in regard to *T. cruzi* stage forms, the life cycle, and the key challenges for the development of effective treatments.

## Target Product Profile

The actual and complex life cycle should lead research into new treatments to achieve a parasitic cure: it reveals the key points that, on the one hand, have made the development of effective drugs and treatments more difficult and, on the other hand, must be the main criteria from now on for the total clearance of the parasite in patients. Tissue tropism (strain-dependent, particularly in the brain and the adipose tissue), genetic diversity, and the challenging morphological forms (quiescent amastigotes) are now recognized as a potential explanation for drug failure and treatment relapse.

In this context, the Chagas Clinical Research Platform (CCRP), mainly launched by the Drugs for Neglected Diseases *initiative* (DND*i*), provided the first target product profile (TPP) for CD in 2009, as well as the revision of alternatives (guidelines, doses, and combination) for using approved drugs.^17^ In short, this TPP stated that ideal drugs had to be active against the acute and chronic stages of CD, with no contraindications, and without genotoxicity, teratogenicity, or pro-arrhythmic potential, among others.

**Figure 2:**
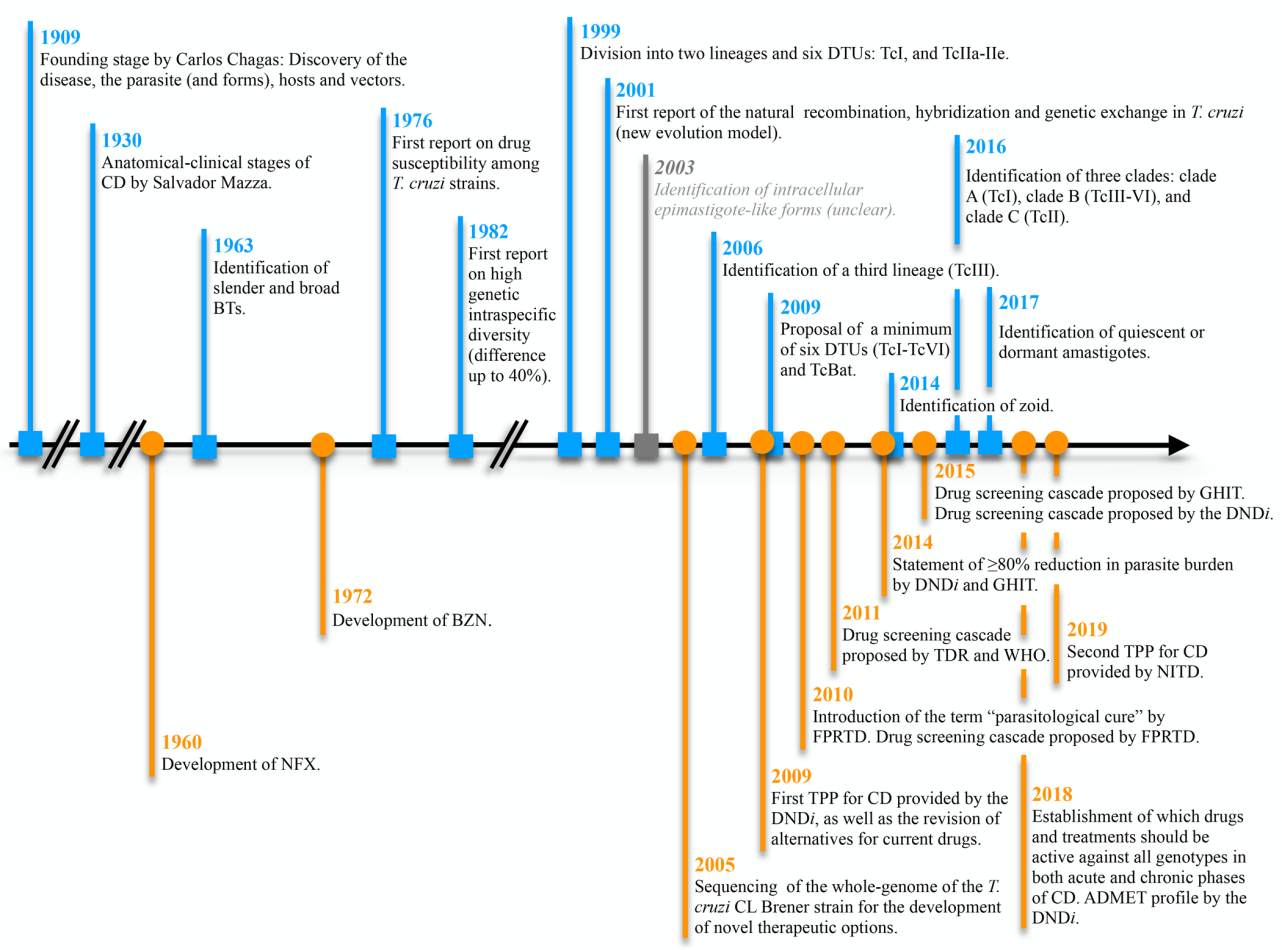
Timeline with the main events in the Chagas disease drug discovery.^17,79−90^ Abbreviations: CD, Chagas disease; BTs, bloodstream trypomastigotes; DTUs, discrete typing units; NFX, nifurtimox; BZN, benznidazole; TPP, Target Product Profile; DND*i*, Drugs for Neglected Diseases *initiative*; FPRTD, Fiocruz Program for Research and Technological Development; TDR, Tropical Diseases Research; WHO, World Health Organization; GHIT, Global Health Innovative Technology; ADMET, absorption, distribution, metabolism, excretion, and tolerability/toxicology; NITD, Norvartis Institute for Tropical Diseases.

However, subsequent findings^7^—genetic diversity, tropism, and life-cycle stages, among others—have led different institutions and experts to provide new TPPs and stringent screening cascades with the aim of achieving a parasitic cure. Some recent examples are listed here. Fiocruz Program for Research and Technological Development (FPRTD) on CD introduced the term “parasitological cure” in 2010, and proposed the first drug-screening cascade for CD drug discovery.^84^ Tropical Diseases Research (TDR) and WHO proposed the second drug-screening cascade for CD in 2011.^85^ DND*i* in 2014,^86^ and Global Health Innovative Technology (GHIT) in 2015,^87^ stated that a treatment should reduce the parasite burden by at least 80%. In 2015, DND*i*^88^ and GHIT^87^ proposed new drug-screening cascades for CD drug discovery. DND*i* established in 2018 that potential drugs and effective treatments should be active against all genotypes and achieve parasitological cure in both the acute and chronic stages of CD, in addition to emphasizing the ADMET profile of potential compounds.^89^ Norvartis Institute for Tropical Diseases (NITD) provided a revisited TPP in 2019. In short, it states that effective treatments should be active in both the acute and chronic stages of CD, in re-infections, and against all DTUs; treatments should eliminate all parasites, including in blood and tissue; and treatments should be safe and well tolerated, with no contraindications nor side effects.^90^ All these events and proposals for drug discovery strategies, together with the main findings regarding both the biology and the genetic background of *T. cruzi*, are summarized in Figure 2.

A new topic to consider is that new drugs should eradicate all parasites in both the blood and the tissues of the host, in order to avoid any relapse after treatment. *T. cruzi* can permeate into the brain, among others, and form nests (dormant amastigotes) in astrocytes.^91^ Spread to the cerebrospinal fluid is a critical point since the blood–brain barrier blocks the passage of most small molecules. Therefore, the requirements for compounds (1) to cross this barrier, (2) to be active against dormant amastigotes, and (3) to be active against all DTUs are the major issues in drug design in order to achieve a parasitic cure. All these points are included in the last TPP.

Given the importance of this, recent years have seen an increase in the publication of numerous viewpoints and reviews discussing future directions in drug discovery for CD.^7,90,92−96^ As new findings have been made, challenges have increased, and both the drug discovery strategy and the TPP need to be modified, as shown in Figure 2.

## Conclusions

CD is still considered a global health problem with significant epidemiological and socioeconomic implications. In the context of this parasitic disease, chemotherapy has presented several drawbacks. Here, we have provided a historical overview on the discoveries—new morphological stages, genetic exchange/genetic diversity, and tropism—around *T. cruzi*, and we have subsequently presented a revised life cycle of the parasite as well as considerations related to the development of effective drugs and treatments to achieve a parasitic cure, that is, the total eradication of the parasite in patients. In recent years, technical advances in several areas, together with changes in research practice and a more propitious funding scenario, have contributed to a better understanding of the biology and life cycle of this parasite, which has made it possible to design the ideal profile of both drugs and therapeutic options for treating CD. Accordingly, many efforts are currently in place to achieve this parasitic cure, developing new drug-screening cascades and new TPPs for CD drug discovery. For this reason, multidisciplinary approaches and combined therapies could resolve this discrepancy and open new directions for finding lead compounds to combat CD. This work aims to be a compilation and an update of previous research, including everything reported in relation to the *T. cruzi* life cycle as well as its key points relevant to the development of effective drugs, with the idea of being a helpful guideline for further research in this area.

## Dedication

We dedicate this work to Prof. Manuel Sánchez Moreno.

## Abbreviations

ADMET: absorption, distribution, metabolism, excretion, and tolerability/toxicology
BT: bloodstream trypomastigote
BZN: benznidazole
CCRP: Chagas Clinical Research Platform
CD: Chagas disease
DND*i*: Drug for Neglected Diseases *initiative*
DTU: discrete typing unit
FPRTD: Fiocruz Program for Research and Technological Development
GHIT: Global Health Innovative Technology
NFX: nifurtimox
NITD: Norvartis Institute for Tropical Diseases
NTD: neglected tropical disease
PV: parasitophorus vacuole
TDR: Tropical Diseases Research
TPP: target product profile
WHO: World Health Organization
